# Global in vitro activity of tigecycline and comparator agents: Tigecycline Evaluation and Surveillance Trial 2004–2013

**DOI:** 10.1186/s12941-015-0085-1

**Published:** 2015-05-10

**Authors:** Daryl J Hoban, Ralf Rene Reinert, Samuel K Bouchillon, Michael J Dowzicky

**Affiliations:** Department of Medical Microbiology & Infectious Disease, University of Manitoba, MS675B-820 Sherbrook Street, R3A 1R9 Winnipeg, Manitoba Canada; Pfizer Vaccines, 23-25, avenue du Dr. Lannelongue, F-75668 Paris Cedex 14, France; IHMA, 2122 Palmer Drive, IL 60173-3817, Schaumburg, USA; Pfizer Inc., 500 Arcola Road; E-Dock, 19426 Collegeville, PA USA

**Keywords:** Antimicrobial drug resistance, Surveillance, Gram-positive bacteria, Gram-negative bacteria, Tigecycline

## Abstract

**Background:**

The Tigecycline Evaluation and Surveillance Trial (TEST) is a global antimicrobial susceptibility surveillance study which has been ongoing since 2004. This report examines the in vitro activity of tigecycline and comparators against clinically important pathogens collected globally between 2004 and 2013.

**Methods:**

Antimicrobial susceptibility was determined using guidelines published by the Clinical and Laboratory Standards Institute. The Cochran Armitage Trend Test was used to identify statistically significant changes in susceptibility between 2004 and 2013.

**Results:**

Among the Enterobacteriaceae susceptibility was highest to the carbapenems [imipenem 97.1% (24,655/25,381), meropenem 97.0% (90,714/93,518)], tigecycline (97.0%, 115,361/118,899) and amikacin (96.9%, 115,200/118,899). Against *Acinetobacter baumannii* the highest rates of susceptibility were for minocycline (84.5%, 14,178/16,778) and imipenem (80.0%, 3,037/3,795). The MIC_90_ for tigecycline was 2 mg/L. 40% (6,743/16,778) of *A. baumannii* isolates were multidrug-resistant. Enterococci were highly susceptible to tigecycline and linezolid (>99%); vancomycin resistance was observed among 2% of *Enterococcus faecalis* (325/14,615) and 35% of *Enterococcus faecium* (2,136/6,167) globally. 40% (14,647/36,448) of *Staphylococcus aureus* were methicillin-resistant while 15% (2,152/14,562) of *Streptococcus pneumoniae* were penicillin-resistant. Against *S. aureus* and *S. pneumoniae* susceptibility to linezolid, vancomycin, and tigecycline was ≥99.9%. Globally, 81% (331/410) of statistically significant susceptibility changes during the study period were decreases in susceptibility.

**Conclusions:**

Amikacin, the carbapenems, and tigecycline were active against most gram-negative pathogens while linezolid, tigecycline, and vancomycin retained activity against most gram-positive pathogens collected in TEST during 2004–2013.

**Electronic supplementary material:**

The online version of this article (doi:10.1186/s12941-015-0085-1) contains supplementary material, which is available to authorized users.

## Introduction

While the initial development of antimicrobial resistance mechanisms may be a local event, antimicrobial resistance has become a problem of global concern, usually resulting in prolonged and expensive therapy [1]. Global travel and migration as well as international trade have blurred the traditional geographical boundaries between countries and continents, enabling the rapid and global spread of resistant organisms [2]. Numerous important resistance mechanisms have shown alarming increases in distribution in recent years, such as extended-spectrum β-lactamases (ESBLs) and carbapenemases [3,4]. This situation is further complicated by the current shortage of new antimicrobial development, increasing the probability that today’s resistant organisms may become tomorrow’s pan-resistant pathogens [5].

The Tigecycline Evaluation and Surveillance Trial (TEST) is a global surveillance study which has been ongoing since 2004. It has been designed specifically to monitor the in vitro activity of the broad-spectrum antimicrobial tigecycline plus comparator antimicrobial agents against numerous clinically important gram-negative and gram-positive organisms. In this report, we examine the in vitro activity of tigecycline and comparators against a collection of gram-negative and -positive organisms collected from medical centres globally between 2004 and 2013. This report updates that of Garrison et al. [6], who examined global antimicrobial susceptibility and resistance rates between 2004 and 2007.

## Materials and methods

Materials and methods for the T.E.S.T. study have been published previously e.g. [7] with minimum inhibitory concentrations (MICs) determined according to the broth microdilution method of the Clinical and Laboratory Standards Institute (CLSI) [8].

After receipt by the central laboratory, International Health Management Associates, Inc. (IHMA, Schaumberg, IL, USA), organism identification confirmation was carried out on all isolates using matrix-assisted laser desorption ionization-time of flight (MALDI-TOF) mass spectrometry (Bruker Daltronics, Bremen, Germany).

Antimicrobial susceptibility was determined using breakpoints approved by the CLSI [9] with the (US) Food and Drug Administration (FDA) breakpoints used for tigecycline [10]. FDA tigecycline breakpoints for *Enterococcus faecalis* (vancomycin-susceptible) were used for all *Enterococcus* isolates in this study and penicillin oral breakpoints (susceptible ≤0.06 mg/L, resistant ≥2 mg/L) were used for *Streptococcus pneumoniae*.

### Multidrug resistance

For the purposes of this manuscript, multidrug-resistant (MDR) was defined as resistance to three or more classes of antimicrobial agents. The classes used to define MDR among the Enterobacteriaceae were aminoglycosides (amikacin), β-lactams (ampicillin, amoxicillin/clavulanate, cefepime, ceftriaxone, or piperacillin-tazobactam), carbapenems (imipenem/meropenem), fluoroquinolones (levofloxacin), glycylcyclines (tigecycline), and tetracyclines (minocycline); the classes used to define MDR *A. baumannii* were aminoglycosides (amikacin), β-lactams (cefepime, ceftazidime, ceftriaxone, or piperacillin-tazobactam), carbapenems (imipenem/meropenem), fluoroquinolones (levofloxacin), and tetracyclines (minocycline); and the classes used to define MDR *P. aeruginosa* were aminoglycosides (amikacin), β-lactams (cefepime, ceftazidime, or piperacillin-tazobactam), carbapenems (imipenem/meropenem), and fluoroquinolones (levofloxacin).

### Statistical analysis

The Cochran Armitage Trend Test was used to identify statistically significant changes in susceptibility between 2004 and 2013. A positive change designated a statistically significant decrease in susceptibility; conversely, a negative change indicated that susceptibility had increased significantly. A statistical significance cut-off value of p < 0.01 was used in this analysis. Imipenem and meropenem data were excluded from the statistical analysis. As previously reported imipenem was replaced by meropenem in 2006 so data for the full period of study were not available for these two antimicrobials. Comparative data from 2004 and 2013 are sometimes presented here to support statistically significant changes in susceptibility; where 2004 and/or 2013 numbers were lower than intervening study years, data from other study years (most often 2005 or 2012) have been presented.

## Results

### Enterobacteriaceae

The Enterobacteriaceae accounted for 118,899 isolates globally between 2004 and 2013. Susceptibility was highest to imipenem (97.1%), meropenem (97.0%), tigecycline (97.0%), and amikacin (96.9%) (Table 1). Globally, statistically significant decreases (p < 0.001–0.0001) in susceptibility were reported against the majority of antimicrobial agents and statistically significant (p < 0.01) changes in susceptibility were observed in all regions (Additional file 1: Table S1).

**Table 1:** **Antimicrobial activity of antimicrobial agents against Enterobacteriaceae collected globally between 2004 - 2013**

	**MIC**_**90**_ **(mg/L)**	**MIC range mg/L**	**% susceptible**	**% resistant**
**Enterobacteriaceae** (n = 118,899)				
AMK	8	≤0.5 – ≥ 128	96.9	1.8
AMC	≥64	≤0.12 – ≥ 64	43.5	46.2
AMP (n = 118,648)	≥64	≤0.5 – ≥ 64	13.0	80.4
FEP	16	≤0.5 – ≥ 64	88.7	8.8
CRO	64	≤0.06 – ≥ 128	72.3	25.7
IPM (n = 25,381)	1	≤0.06 – ≥ 32	97.1	0.9
LVX	≥16	≤0.008 – ≥ 16	79.0	18.4
MEM (n = 93,518)	0.25	≤0.06 – ≥ 32	97.0	2.3
MIN	16	≤0.5 – ≥ 32	74.9	13.1
TZP	64	≤0.06 – ≥ 256	84.0	9.4
TGC	1	≤0.008 – ≥ 32	97.0	0.6
**Enterobacteriaceae**, MDR (n = 9,372)				
AMK	≥128	≤0.5 – ≥ 128	75.4	17.8
AMC	≥64	0.25 – ≥ 64	11.8	67.8
AMP (n = 9,371)	≥64	≤0.5 – ≥ 64	0.2	99.5
FEP	≥64	≤0.5 – ≥ 64	47.3	44.2
CRO	≥128	≤0.06 – ≥ 128	20.3	78.2
IPM (n = 1,158)	4	≤0.06 – ≥ 32	85.2	12.1
LVX	≥16	≤0.008 – ≥ 16	6.1	92.1
MEM (n = 8,214)	≥32	≤0.06 – ≥ 32	75.3	21.8
MIN	≥32	≤0.5 – ≥ 32	12.0	81.5
TZP	≥256	≤0.06 – ≥ 256	39.5	44.0
TGC	4	≤0.008 – ≥ 32	83.2	6.9

AMK, amikacin; AMC, amoxicillin-clavulanate; AMP, ampicillin; BL, β-lactamase; Car-R, carbapenem-resistant; FEP, cefepime; CAZ, ceftazidime; CRO, ceftriaxone; IPM, imipenem; LVX, levofloxacin; LZD, linezolid; MDR, multidrug-resistant; MEM, meropenem; MIN, minocycline; PEN, penicillin; TZP, piperacillin-tazobactam; TGC, tigecycline; VAN, vancomycin.

Among MDR Enterobacteriaceae (n = 9,372; Table 1), statistically significant changes in susceptibility were observed in all regions excluding the Middle East; most of these changes (20/28) represented decreases in susceptibility (Additional file 1: Table S1).

### *Enterobacter* spp

A total of 32,987 isolates of *Enterobacter* spp. were collected between 2004 and 2013 (Table 2). High (>95%) levels of susceptibility were observed for amikacin, imipenem, meropenem, and tigecycline. Among the 578 carbapenem-resistant (Car-R) isolates, the highest susceptibility rate was reported for tigecycline (83.0%).

**Table 2:** **Antimicrobial activity of antimicrobial agents against members of the Enterobacteriaceae collected globally between 2004 - 2013**

	**MIC**_**90**_ **(mg/L)**	**MIC range mg/L**	**% susceptible**	**% resistant**
***Enterobacter*** **spp.** (n = 32,987)				
AMK	4	≤0.5– ≥ 128	97.2	1.8
FEP	8	≤0.5– ≥ 64	91.7	5.8
CRO	64	≤0.06– ≥ 128	61.7	35.1
IPM (n = 6,963)	1	≤0.06– ≥ 32	95.7	1.1
LVX	8	≤0.008– ≥ 16	86.8	10.7
MEM (n = 26,024)	0.25	≤0.06– ≥ 32	97.2	1.9
MIN	16	≤0.5– ≥ 32	73.7	12.5
TZP	128	≤0.06– ≥ 256	76.1	12.1
TGC	2	≤0.008– ≥ 32	95.4	1.1
***Enterobacter*** **spp.**, Car-R (n = 578)				
AMK	≥128	≤0.5– ≥ 128	68.3	23.7
FEP	≥64	≤0.5– ≥ 64	35.6	52.2
CRO	≥128	≤0.06– ≥ 128	6.7	91.9
LVX	≥16	≤0.008– ≥ 16	39.1	54.0
MIN	≥32	≤0.5– ≥ 32	39.8	38.8
TZP	≥256	≤0.06– ≥ 256	22.5	60.7
TGC	4	0.06–16	83.0	5.2
***Escherichia coli*** (n = 37,038)				
AMK	8	≤0.5– ≥ 128	98.3	0.9
AMC	32	≤0.12– ≥ 64	66.6	14.2
AMP	≥64	≤0.5– ≥ 64	35.6	63.5
FEP	16	≤0.5– ≥ 64	88.5	9.0
CRO	64	≤0.06– ≥ 128	79.1	19.9
IPM (n = 8,091)	0.5	≤0.06– ≥ 32	99.4	0.3
LVX	≥16	≤0.008– ≥ 16	66.1	31.5
MEM (n = 28,947)	≤0.06	≤0.06– ≥ 32	99.1	0.5
MIN	16	≤0.5– ≥ 32	77.0	13.0
TZP	16	≤0.06– ≥ 256	91.3	4.4
TGC	0.5	≤0.008– ≥ 32	>99.9	<0.1
***Escherichia coli***, ESBL-positive (n = 5,178)				
AMK	16	≤0.5– ≥ 128	94.2	2.7
AMC	32	0.25– ≥ 64	31.3	28.5
AMP	≥64	1– ≥ 64	0.4	99.5
FEP	≥64	≤0.5– ≥ 64	32.4	54.8
CRO	≥128	≤0.06– ≥ 128	1.3	97.5
IPM (n = 643)	0.5	≤0.06–8	98.1	0.6
LVX	≥16	≤0.008– ≥ 16	18.1	78.5
MEM (n = 4,535)	0.12	≤0.06– ≥ 32	98.5	0.8
MIN	≥32	≤0.5– ≥ 32	63.2	22.7
TZP	64	≤0.06– ≥ 256	77.0	9.9
TGC	0.5	≤0.008–8	>99.9	<0.1
***Escherichia coli***, Car-R (n = 181)				
AMK	≥128	≤0.5– ≥ 128	50.8	38.1
AMC	≥64	2– ≥ 64	11.6	76.8
AMP	≥64	1– ≥ 64	8.3	90.6
FEP	≥64	≤0.5– ≥ 64	28.2	56.4
CRO	≥128	≤0.06– ≥ 128	9.9	90.1
LVX	≥16	≤0.008– ≥ 16	22.7	71.3
MIN	≥32	≤0.5– ≥ 32	43.1	35.4
TZP	≥256	1– ≥ 256	29.3	55.8
TGC	2	≤0.008–16	97.2	1.7
***Klebsiella oxytoca*** (n = 6,940)				
AMK	4	≤0.5– ≥ 128	98.8	0.8
AMC	32	0.25– ≥ 64	79.4	12.4
FEP	2	≤0.5– ≥ 64	96.1	2.4
CRO	16	≤0.06– ≥ 128	81.5	16.2
IPM (n = 1,454)	0.5	≤0.06– ≥ 32	99.2	0.5
LVX	2	≤0.008– ≥ 16	92.1	5.7
MEM (n = 5,486)	0.12	≤0.06– ≥ 32	98.8	0.8
MIN	8	≤0.5– ≥ 32	88.2	5.6
TZP	≥256	≤0.06– ≥ 256	85.1	12.5
TGC	1	≤0.008–16	98.7	0.2
***Klebsiella pneumoniae*** (n = 28,928)				
AMK	16	≤0.5– ≥ 128	94.4	3.1
AMC	32	≤0.12– ≥ 64	67.9	20.2
FEP	≥64	≤0.5– ≥ 64	81.0	15.8
CRO	≥128	≤0.06– ≥ 128	70.1	28.8
IPM (n = 6,008)	0.5	≤0.06– ≥ 32	97.7	1.6
LVX	≥16	≤0.008– ≥ 16	77.4	19.5
MEM (n = 22,920)	0.25	≤0.06– ≥ 32	93.5	5.4
MIN	16	≤0.5– ≥ 32	71.4	18.0
TZP	≥256	≤0.06– ≥ 256	79.4	14.9
TGC	2	≤0.008– ≥ 32	95.3	0.8
***Klebsiella pneumoniae***, ESBL-positive (n = 5,899)				
AMK	32	≤0.5– ≥ 128	83.6	9.5
AMC	≥64	≤0.12– ≥ 64	18.3	48.3
FEP	≥64	≤0.5– ≥ 64	31.9	56.6
CRO	≥128	≤0.06– ≥ 128	1.4	97.0
IPM (n = 946)	1	≤0.06– ≥ 32	91.3	6.1
LVX	≥16	≤0.008– ≥ 16	35.2	57.1
MEM (n = 4,953)	2	≤0.06– ≥ 32	88.3	9.0
MIN	≥32	≤0.5– ≥ 32	48.4	34.5
TZP	≥256	≤0.06– ≥ 256	44.3	39.2
TGC	2	≤0.008– ≥ 32	92.3	1.6
***Klebsiella pneumoniae***, Car-R (n = 1,330)				
AMK	≥128	≤0.5– ≥ 128	57.5	18.4
AMC	≥64	1– ≥ 64	1.7	96.5
FEP	≥64	≤0.5– ≥ 64	7.4	85.0
CRO	≥128	≤0.06– ≥ 128	1.8	97.5
LVX	≥16	≤0.008– ≥ 16	10.1	85.9
MIN	≥32	≤0.5– ≥ 32	52.2	25.3
TZP	≥256	0.12– ≥ 256	4.7	91.3
TGC	2	0.03–16	92.0	2.1
***Klebsiella pneumoniae***, ESBL-positive, Car-R (n = 505)				
AMK	≥128	≤0.5– ≥ 128	59.8	15.6
AMC	≥64	8– ≥ 64	0.8	96.2
FEP	≥64	1– ≥ 64	4.8	83.6
CRO	≥128	2– ≥ 128	0.0	99.6
LVX	≥16	0.015– ≥ 16	6.7	88.5
MIN	≥32	≤0.5– ≥ 32	49.7	28.5
TZP	≥256	1– ≥ 256	2.0	92.9
TGC	2	0.12–16	91.1	3.6
***Serratia marcescens*** (n = 13,006)				
AMK	8	≤0.5– ≥ 128	96.5	2.1
AMC	≥64	≤0.12– ≥ 64	3.3	93.9
AMP (n = 12,963)	≥64	≤0.5– ≥ 64	2.7	88.0
FEP	2	≤0.5– ≥ 64	95.2	3.5
CRO	16	≤0.06– ≥ 128	79.6	16.9
IPM (n = 2,865)	1	≤0.06–8	92.2	1.2
LVX	2	≤0.008– ≥ 16	92.9	4.4
MEM (n = 10,141)	0.25	≤0.06– ≥ 32	97.4	1.9
MIN	8	≤0.5– ≥ 32	72.3	8.5
TZP	16	≤0.06– ≥ 256	92.4	3.2
TGC	2	≤0.008– ≥ 32	95.8	0.7
***Serratia marcescens***, Car-R (n = 229)				
AMK	≥128	≤0.5– ≥ 128	67.7	24.9
AMC	≥64	≤0.12– ≥ 64	0.4	99.1
AMP	≥64	16– ≥ 64	0.0	95.2
FEP	≥64	≤0.5– ≥ 64	51.1	39.3
CRO	≥128	≤0.06– ≥ 128	26.2	70.3
LVX	≥16	0.03– ≥ 16	60.7	31.0
MIN	16	≤0.5– ≥ 32	54.6	26.6
TZP	≥256	0.25– ≥ 256	45.4	41.0
TGC	4	0.12–16	83.8	4.8

AMK, amikacin; AMC, amoxicillin-clavulanate; AMP, ampicillin; BL, β-lactamase; Car-R, carbapenem-resistant; FEP, cefepime; CAZ, ceftazidime; CRO, ceftriaxone; ESBL, extended-spectrum β-lactamase; IPM, imipenem; LVX, levofloxacin; LZD, linezolid; MEM, meropenem; MIN, minocycline; PEN, penicillin; TZP, piperacillin-tazobactam; TGC, tigecycline; VAN, vancomycin.

Statistically significant decreases in global susceptibility were recorded to cefepime, ceftriaxone, minocycline, and piperacillin-tazobactam (each p < 0.0001) between 2004 and 2013 (Additional file 1: Table S1); minocycline global susceptibility declined between 2004 (85.5%) and 2011 (57.6%) but increased to 84.5% in 2012 and 86.1% in 2013 (data not shown). Tigecycline susceptibility increased in North America (p < 0.0001; 93.7% in 2004 and 96.4% in 2013) but decreased in Latin America [p < 0.01; 98.2% in 2004 and 83.3% in 2013 (n = 24 in 2013)].

#### Escherichia coli

Of 37,038 *E. coli* isolates collected globally, most were susceptible to tigecycline (>99.9%), imipenem (99.4%), meropenem (99.1%), and amikacin (98.3%) (Table 2). Carbapenem-resistant *E. coli* accounted for only 0.5% (181/37,038) of isolates globally (Table 2) but reached 2.0% (13/661) in Africa (data not shown); tigecycline susceptibility remained high (97.2%) among these resistant isolates.

Regionally, statistically significant decreases in susceptibility were most prevalent in Asia/Pacific Rim, North America, and Latin America (Additional file 1: Table S1). Tigecycline susceptibility decreased significantly (p < 0.01) in North America and globally, although the actual susceptibility rates decreased from 100% to 99.6% and from 100% to 99.8%, respectively, between 2004 and 2013. Among carbapenem-resistant *E. coli*, global susceptibility decreased to cefepime (p < 0.0001; 42.9% in 2006 and 0.0% in 2013), ceftriaxone (p < 0.01; 16.7% in 2004 and 0.0% in 2013), and tigecycline (p < 0.01; 100% in 2004 and 80.0% in 2013), although only 181 isolates were identified between 2004 and 2013.

ESBL production was observed among 5,178 (14.0%) *E. coli* isolates globally (Table 2), ranging from 5.0% (650/12,934) in North America to 24.7% (1,049/4,239) in Latin America and 25.9% (330/1,273) in Middle East (data not shown). Global ESBL percentages significantly (p < 0.0001) increased from 5.8% (148/2,549) in 2004 to 15.5% (266/1,712) in 2013, reaching a maximum of 18.8% (911/4,858) in 2012. Amikacin, imipenem, meropenem, and tigecycline susceptibility remained high (>94%) among ESBL-positive isolates.

#### Klebsiella oxytoca

Globally, *K. oxytoca* (n = 6,940) were highly susceptible (>96%) to amikacin, cefepime, imipenem, meropenem, and tigecycline (Table 2). In Asia/Pacific Rim, statistically significant decreases in amikacin, minocycline (each p < 0.0001), and levofloxacin (p < 0.01) susceptibility were observed; globally, piperacillin-tazobactam susceptibility decreased significantly (p < 0.01) while tigecycline susceptibility increased significantly (p < 0.01) from 98.6% in 2004 to 99.4% in 2013 (Additional file 1: Table S1). Only 301 ESBL-producing *K. oxytoca* isolates were identified globally between 2004 and 2013; totals ranged from 5 in Africa to 136 in Europe.

#### Klebsiella pneumoniae

A total of 28,928 isolates of *K. pneumoniae* were collected globally between 2004 and 2013. Susceptibility was highest to imipenem (97.7%), tigecycline (95.3%), amikacin (94.4%), and meropenem (93.5%) (Table 2). After gradual declines in susceptibility between 2004 and 2011, susceptibility to several antimicrobials increased between 2011 and 2013 (cefepime, susceptibility increased by 5.8%; levofloxacin, by 8.7%; ceftriaxone, by 8.9%; amoxicillin-clavulanate, by 9.7%; piperacillin-tazobactam, by 14.3%; and minocycline, by 22.9%). Carbapenem resistance was observed in 1330 (4.6%) isolates, with the agent with the highest susceptibility against this subset of resistant isolates being tigecycline (92.0% susceptible); 505 ESBL-positive isolates were also carbapenem-resistant (Table 2).

Statistically significant reductions in susceptibility among *K. pneumoniae* were noted to most antimicrobials globally as well as in Asia/Pacific Rim and Europe (Additional file 1: Table S1). Carbapenem-resistant *K. pneumoniae* demonstrated low (≤10.1%) and significantly decreased susceptibility to amoxicillin-clavulanate, cefepime, ceftriaxone, levofloxacin, and piperacillin-tazobactam globally (Additional file 1: Table S1).

ESBL production, observed in 5,899 (20.4%) *K. pneumoniae* isolates globally, was highest in Africa (42.8%, 249/582) and lowest in North America (9.5%, 983/10,366). Susceptibility among these isolates was highest to tigecycline (92.3%) and imipenem (91.3%) (Table 2). Global ESBL percentages increased significantly (p < 0.0001) from 13.9% (289/2086) in 2004 to 18.5% (227/1229) in 2013, achieving a maximum of 25.6% (757/2,954) in 2011.

Statistically significant decreases in antimicrobial susceptibility were reported globally among ESBL-positive *K. pneumoniae* for cefepime and minocycline (both p < 0.0001) as well as ceftriaxone (p < 0.001), while increases in susceptibility were recorded to amikacin (p < 0.0001) and piperacillin-tazobactam (p < 0.01) (Additional file 1: Table S1).

#### Serratia marcescens

Globally, a total of 13,006 *S. marcescens* isolates were collected. Susceptibility was high (>92%) to most agents on the panel with the exceptions of ampicillin (2.7%), amoxicillin-clavulanate (3.3%), minocycline (72.3%), and ceftriaxone (79.6%). Carbapenem resistance was observed in 229 (1.8%) isolates (Table 2).

Minocycline susceptibility decreased significantly globally (p < 0.0001; 88.8% in 2004 and 85.1% in 2013) and in all regions bar Middle East (p < 0.0001; Additional file 1: Table S1); minocycline susceptibility reached a global minimum of 48.1% in 2009. Tigecycline susceptibility decreased significantly in Asia/Pacific Rim (p < 0.001; 96.8% in 2004 and 92.5% in 2011) and Latin America (p < 0.01; 100% in 2004 and 87.0% in 2012) (Additional file 1: Table S1).

#### Acinetobacter baumannii

The highest levels of in vitro susceptibility against *A. baumannii* isolates (n = 16,778) in this study were reported for minocycline (84.5%) and imipenem (80.0%) (Table 3). No breakpoints are available for tigecycline, for which a MIC_90_ of 2 mg/L was observed. More than 40% (6,743/16,778) of *A. baumannii* isolates were MDR globally (Table 3), with rates highest in Africa (59.4%, 202/340), Middle East (67.2%, 452/673), and Latin America (67.8%, 1,388/2,048) (data not shown); the highest levels of susceptibility against these MDR isolates was observed for minocycline (70.3% susceptible), while tigecycline retained a MIC_90_ of 2 mg/L (Table 3).

**Table 3:** **Antimicrobial activity of antimicrobial agents against** ***Acinetobacter baumannii*** **,** ***Haemophilus influenzae*** **and** ***Pseudomonas aeruginosa*** **collected globally between 2004 - 2013**

	**MIC**_**90**_ **(mg/L)**	**MIC range mg/L**	**% susceptible**	**% resistant**
***Acinetobacter baumannii*** (n = 16,778)				
AMK	≥128	≤0.5 – ≥ 128	61.1	32.4
AMC	≥64	≤0.12 – ≥ 64	-	-
AMP	≥64	≤0.5 – ≥ 64	-	-
FEP	≥64	≤0.5 – ≥ 64	44.3	42.9
CAZ	≥64	≤8 – ≥ 64	41.2	52.1
CRO	≥128	≤0.06 – ≥ 128	23.3	54.6
IPM (n = 3,795)	≥32	≤0.06 – ≥ 32	80.0	16.8
LVX	≥16	≤0.008 – ≥ 16	43.0	46.6
MEM (n = 12,983)	≥32	≤0.06 – ≥ 32	54.8	41.1
MIN	8	≤0.5 – ≥ 32	84.5	5.1
TZP	≥256	≤0.06 – ≥ 256	42.9	47.5
TGC	2	≤0.008 – ≥ 32	-	-
***Acinetobacter baumannii***, MDR (n = 6,743)				
AMK	≥128	≤0.5 – ≥ 128	18.4	74.1
AMC	≥64	1 – ≥ 64	-	-
AMP	≥64	≤0.5 – ≥ 64	-	-
FEP	≥64	≤0.5 – ≥ 64	5.5	80.1
CAZ	≥64	≤8 – ≥ 64	4.0	90.8
CRO	≥128	≤0.06 – ≥ 128	0.7	94.6
IPM (n = 896)	≥32	0.25 – ≥ 32	30.9	64.6
LVX	≥16	0.03 – ≥ 16	2.4	89.3
MEM (n = 5,847)	≥32	≤0.06 – ≥ 32	11.9	84.0
MIN	16	≤0.5 – ≥ 32	70.3	11.3
TZP	≥256	≤0.06 – ≥ 256	3.0	90.5
TGC	2	≤0.008 – ≥ 32	-	-
***Haemophilus influenzae*** (n = 15,925)				
AMK	8	≤0.5 – ≥ 128	-	-
AMC	1	≤0.12 – ≥ 64	99.7	0.3
AMP	32	≤0.5 – ≥ 64	78.3	19.3
FEP	≤0.5	≤0.5 – ≥ 64	99.5	-
CRO	≤0.06	≤0.06 – 32	99.9	-
IPM (n = 3,672)	1	≤0.06 – ≥ 32	99.9	-
LVX	0.03	≤0.008 – ≥ 16	99.9	-
MEM (n = 12,253)	0.12	≤0.06 – 2	99.9	-
MIN	1	≤0.5 – ≥ 32	98.6	0.5
TZP	≤0.06	≤0.06 – 64	99.8	0.2
TGC	0.25	≤0.008 – 4	98.9	-
***Haemophilus influenzae***, BL-pos (n = 3,207)				
AMK	8	≤0.5 – ≥ 128	-	-
AMC	2	≤0.12 – ≥ 64	98.9	1.1
AMP	≥64	≤0.5 – ≥ 64	0.2	95.2
FEP	≤0.5	≤0.5 – ≥ 64	99.1	-
CRO	≤0.06	≤0.06 – 16	99.9	-
IPM (n = 803)	1	≤0.06 – 4	100	-
LVX	0.03	≤0.008 – ≥ 16	99.8	-
MEM (n = 2,404)	0.12	≤0.06 – 2	99.8	-
MIN	1	≤0.5 – ≥ 32	98.4	0.5
TZP	≤0.06	≤0.06 – 64	99.7	0.3
TGC	0.25	≤0.008 – 1	99.0	-
***Pseudomonas aeruginosa*** (n = 28,413)				
AMK	16	≤0.5 – ≥ 128	90.2	6.5
AMC	≥64	≤0.12 – ≥ 64	-	-
AMP	≥64	≤0.5 – ≥ 64	-	-
FEP	32	≤0.5 – ≥ 64	74.3	13.5
CAZ	32	≤8 – ≥ 64	74.0	18.1
CRO	≥128	≤0.06 – ≥ 128	-	-
IPM (n = 6,303)	8	≤0.06 – ≥ 32	76.4	17.5
LVX	≥16	≤0.008 – ≥ 16	63.9	29.5
MEM (n = 22,110)	16	≤0.06 – ≥ 32	70.3	22.3
MIN	≥32	≤0.5 – ≥ 32	-	-
TZP	128	≤0.06 – ≥ 256	73.1	15.3
TGC	16	≤0.008 – ≥ 32	-	-
***Pseudomonas aeruginosa***, MDR (n = 3,496)				
AMK	≥128	≤0.5 – ≥ 128	46.3	43.5
AMC	≥64	1 – ≥ 64	-	-
AMP	≥64	≤0.5 – ≥ 64	-	-
FEP	≥64	≤0.5 – ≥ 64	8.2	69.0
CAZ	≥64	≤8 – ≥ 64	11.7	77.7
CRO	≥128	2 – ≥ 128	-	-
IPM (n = 557)	≥32	0.5 – ≥ 32	5.9	92.1
LVX	≥16	0.03 – ≥ 16	2.1	96.3
MEM (n = 2,939)	≥32	≤0.06 – ≥ 32	4.9	91.8
MIN	≥32	≤0.5 – ≥ 32	-	-
TZP	≥256	0.25 – ≥ 256	10.9	67.7
TGC	≥32	≤0.008 – ≥ 32	-	-

AMK, amikacin; AMC, amoxicillin-clavulanate; AMP, ampicillin; BL, β-lactamase; Car-R, carbapenem-resistant; FEP, cefepime; CAZ, ceftazidime; CRO, ceftriaxone; IPM, imipenem; LVX, levofloxacin; LZD, linezolid; MDR, multidrug-resistant; MEM, meropenem; MIN, minocycline; PEN, penicillin; TZP, piperacillin-tazobactam; TGC, tigecycline; VAN, vancomycin.

Significant decreases in susceptibility were observed to all antimicrobials with available breakpoints globally (all p < 0.0001) (Additional file 1: Table S1). Among MDR *A. baumannii*, significant reductions in global susceptibility were noted to minocycline, and piperacillin-tazobactam (p < 0.0001). Significant increases in MDR *A. baumannii* susceptibility were observed to amikacin (p < 0.001) in Latin America, although susceptibility was only 8.8% during the complete study interval, and to minocycline (p < 0.001) in Africa.

#### Haemophilus influenzae

A total of 15,925 isolates of *H. influenzae* were collected globally. All isolates were highly susceptible (>98.5%) to the antimicrobial agents on the panel with the exception of ampicillin (78.3% susceptibility) (Table 3).

Globally, increased ampicillin susceptibility was recorded (p < 0.001; 76.3% in 2004 and 80.2% in 2012) while decreased susceptibility was observed to ceftriaxone (p < 0.0001; 100% in 2004 and 99.7% in 2013) and tigecycline (p < 0.0001; 100% in 2004 and 95.4% in 2011 [100% in 2013]) (Additional file 1: Table S1). Significant decreases in tigecycline susceptibility were recorded in Asia/Pacific Rim (p < 0.001; 100% in 2004 and 92.7% in 2012) and Latin America (p < 0.01; 100% in 2004 and 93.6% in 2012).

β-lactamase production was reported in 20.1% (3,207/15,925) of *H. influenzae* isolates globally; these isolates retained high susceptibility to most agents with the exception of ampicillin (0.2% susceptible; Table 3). No statistically significant changes in susceptibility were seen among β-lactamase-positive *H. influenzae*.

#### Pseudomonas aeruginosa

Globally, 28,413 *P. aeruginosa* isolates were collected between 2004 and 2013. These isolates showed a high susceptibility to amikacin (90.2%; Table 3). Multidrug resistance was reported among 12.3% (3,496/28,413) of *P. aeruginosa* isolates.

Globally, statistically significant decreases in amikacin (p < 0.01; 94.9% in 2004 and 88.5% in 2011 [95.2% in 2013]), ceftazidime (p < 0.01; 79.4% in 2004 and 68.9% in 2011 [82.4% in 2013]), and piperacillin-tazobactam (p < 0.0001; 78.5% in 2004 and 67.4% in 2011 [81.1% in 2013]) susceptibility were observed among *P. aeruginosa* (Additional file 1: Table S1). Susceptibility to several antimicrobial agents increased between 2011 and 2013: amikacin (by 6.7%), cefepime (7.6%), ceftazidime (13.5%), and piperacillin-tazobactam (13.7%).

#### Enterococcus faecalis

Global susceptibility among *E. faecalis* isolates (n = 14,615) was highest to linezolid (99.8%), tigecycline (99.7%), ampicillin (99.4%), and penicillin (99.2%) (Table 4).

**Table 4:** **Antimicrobial activity of antimicrobial agents against** ***Enterococcus*** **and** ***Staphylococcus*** **spp. collected globally between 2004 - 2013**

	**MIC**_**90**_ **(mg/L)**	**MIC range (mg/L)**	**% susceptible**	**% resistant**
***Enterococcus faecalis*** (n = 14,615)				
AMC	1	≤0.03 – ≥ 16	-	-
AMP	2	≤0.06 – ≥ 32	99.4	0.6
CRO	≥128	≤0.03 – ≥ 128	-	-
IPM (n = 3,208)	4	≤0.12 – ≥ 32	-	-
LVX	≥64	≤0.06 – ≥ 64	64.9	33.9
LIN	2	≤0.5 – ≥ 16	99.8	<0.1
MEM (n = 11,407)	8	≤0.12 – ≥ 32	-	-
MIN	≥16	≤0.25 – ≥ 16	34.8	28.2
PEN	4	≤0.06 – ≥ 16	99.2	0.8
TZP	8	≤0.25 – ≥ 32	-	-
TGC	0.25	≤0.008 – 2	99.7	-
VAN	2	≤0.12 – ≥ 64	97.5	2.2
***Enterococcus faecalis***, VR (n = 325)				
AMC	2	0.06 – ≥ 16	-	-
AMP	4	0.12 – ≥ 32	94.5	5.5
CRO	≥128	≤0.03 – ≥ 128	-	-
IPM (n = 98)	8	≤0.12 – ≥ 32	-	-
LVX	≥64	0.5 – ≥ 64	4.9	93.5
LIN	2	≤0.5 – ≥ 16	98.5	1.2
MEM (n = 227)	≥32	≤0.12 – ≥ 32	-	-
MIN	≥16	≤0.25 – ≥ 16	44.3	17.8
PEN	8	0.25 – ≥ 16	94.5	5.5
TZP	16	0.5 – ≥ 32	-	-
TGC	0.25	≤0.008 – 1	97.8	-
***Enterococcus faecium*** (n = 6,167)				
AMC	≥16	≤0.03 – ≥ 16	-	-
AMP	≥32	≤0.06 – ≥ 32	15.6	84.4
CRO	≥128	≤0.03 – ≥ 128	-	-
IPM (n = 1,162)	≥32	≤0.12 – ≥ 32	-	-
LVX	≥64	≤0.06 – ≥ 64	13.1	83.6
LIN	2	≤0.5 – ≥ 16	99.2	0.3
MEM (n = 5,005)	≥32	≤0.12 – ≥ 32	-	-
MIN	≥16	≤0.25 – ≥ 16	68.5	14.3
PEN	≥16	≤0.06 – ≥ 16	15.6	84.4
TZP	≥32	≤0.25 – ≥ 32	-	-
TGC	0.25	≤0.008 – 4	99.7	-
VAN	≥64	≤0.12 – ≥ 64	64.0	34.6
***Enterococcus faecium***, VR (n = 2,136)				
AMC	≥16	≤0.03 – ≥ 16	-	-
AMP	≥32	≤0.06 – ≥ 32	1.5	98.5
CRO	≥128	≤0.03 – ≥ 128	-	-
IPM (n = 556)	≥32	0.5 – ≥ 32	-	-
LVX	≥64	0.5 – ≥ 64	0.8	98.8
LIN	2	≤0.5 – ≥ 16	98.4	0.7
MEM (n = 1,580)	≥32	≤0.12 – ≥ 32	-	-
MIN	≥16	≤0.25 – ≥ 16	65.0	13.3
PEN	≥16	≤0.06 – ≥ 16	1.7	98.3
TZP	≥32	≤0.25 – ≥ 32	-	-
TGC	0.12	≤0.008 – 4	99.2	-
***Staphylococcus aureus*** (n = 36,448)				
AMC	≥16	≤0.03 – ≥ 16	-	-
AMP	≥32	≤0.06 – ≥ 32	-	-
CRO	≥128	≤0.03 – ≥ 128	-	-
IPM (n = 7,302)	16	≤0.12 – ≥ 32	-	-
LVX	32	≤0.06 – ≥ 64	64.1	34.3
LIN	4	≤0.5 – ≥ 16	>99.9	<0.1
MEM (n = 29,146)	16	≤0.12 – ≥ 32	-	-
MIN	0.5	≤0.25 – ≥ 16	97.3	0.9
PEN	≥16	≤0.06 – ≥ 16	10.9	89.1
TZP	≥32	≤0.25 – ≥ 32	-	-
TGC	0.25	≤0.008 – 1	99.9	-
VAN	1	≤0.12 – 4	100	0
***Staphylococcus aureus***, MRSA (n = 14,647)				
AMC	≥16	≤0.03 – ≥ 16	-	-
CRO	≥128	≤0.03 – ≥ 128	-	-
IPM (n = 3,235)	≥32	≤0.12 – ≥ 32	-	-
LVX	≥64	≤0.06 – ≥ 64	22.1	75.9
LIN	2	≤0.5 – ≥ 16	99.9	<0.1
MEM (n = 11,412)	≥32	≤0.12 – ≥ 32	-	-
MIN	2	≤0.25 – ≥ 16	94.7	1.7
TZP	≥32	≤0.25 – ≥ 32	-	-
TGC	0.25	≤0.008 – 1	99.9	-
VAN	1	≤0.12 – 4	99.9	0

AMK, amikacin; AMC, amoxicillin-clavulanate; AMP, ampicillin; AZM, azithromycin; FEP, cefepime; CAZ, ceftazidime; CLR, clarithromycin; CLI, clindamycin; CRO, ceftriaxone; ERY, erythromycin; IPM, imipenem; LVX, levofloxacin; LZD, linezolid; MEM, meropenem; MIN, minocycline; MRSA, methicillin-resistant *S. aureus*; TZP, piperacillin-tazobactam; TGC, tigecycline; VAN, vancomycin; VR, vancomycin-resistant.

Statistically significant reductions in ampicillin, minocycline, and penicillin susceptibility (each p < 0.0001) were seen in Europe, North America, and globally; susceptibility to levofloxacin (p < 0.0001) and vancomycin (p < 0.01) increased globally (Additional file 2: Table S2). Minocycline susceptibility among *E. faecalis* decreased from 41.8% in 2004 to 26.6% in 2010 but subsequently increased to 36.7% in 2013.

Vancomycin resistance was observed among 325 isolates (2.2%) of *E. faecalis* globally; these resistant isolates were highly susceptible to linezolid, tigecycline, ampicillin, and penicillin (≥94.5%) (Table 4). Among vancomycin-resistant (VR) isolates, ampicillin, minocycline, and penicillin susceptibility decreased significantly lobally (p < 0.0001, p < 0.0001 and p < 0.001, respectively) and in North America (each p < 0.0001) (Additional file 2: Table S2).

#### Enterococcus faecium

In total, 6,167 isolates of *E. faecium* were submitted globally. Susceptibility was highest to tigecycline (99.7%) and linezolid (99.2%). Among all *E. faecium*, 2,136 (34.6%) isolates were vancomycin-resistant (Table 4).

Tigecycline susceptibility in North America decreased significantly (p < 0.01), from 100% in 2004 to 97.9% in 2011 before returning to 100% in 2013; decreased levofloxacin (p < 0.0001) and ampicillin (p < 0.01) susceptibility were observed in Europe, while decreases in minocycline (p < 0.0001) and penicillin (p < 0.01) susceptibility occurred in Asia/Pacific Rim (Additional file 2: Table S2).

Globally, VR *E. faecium* isolates (n = 2,136) were highly susceptible to tigecycline and linezolid (99.2% and 98.4% susceptible, respectively) (Table 4). Global vancomycin resistance decreased from 47.4% in 2004 (188/397) to 37.2% in 2013 (121/325), reaching a minimum of 25.8% in 2010 (217/840). Regionally, vancomycin resistance ranged from 12.5% (356/2,844) in Europe to 66.8% (1,438/2,152) in North America.

A global decrease in minocycline susceptibility (p < 0.0001) was observed among VR *E. faecium* isolates. Tigecycline susceptibility among VR *E. faecium* decreased significantly (p < 0.01) in North America, although susceptibility through the 2004–2013 period was high (99.2%; Additional file 2: Table S2).

#### Staphylococcus aureus

A total of 36,448 isolates of *S. aureus* were contributed globally and susceptibility was highest to linezolid, vancomycin, and tigecycline (≥99.9%), while 97.3% of isolates were susceptible to minocycline (Table 4).

Levofloxacin susceptibility among *S. aureus* decreased in Asia/Pacific Rim (p < 0.0001; 68.3% in 2004 and 27.9% in 2012) and Europe (p < 0.0001; 71.5% in 2004 and 66.4% in 2012) but increased in North America (p < 0.0001; 53.0% in 2004 and 62.4% in 2013) and globally (p < 0.0001; 59.5% in 2004 and 65.2% in 2013). Minocycline susceptibility increased in Middle East (p < 0.0001; 76.5% in 2005 and 98.6% in 2013) but decreased in Asia/Pacific Rim (p < 0.0001; 88.8% in 2004 and 62.1% in 2010 [97.1% in 2012]), Latin America (p < 0.0001; 98.5% in 2004 and 94.1% in 2011 [98.1% in 2012]), and globally (p < 0.01; 98.6% in 2004 and 94.8% in 2010 [99.2% in 2013]) (Additional file 2: Table S2).

More than 40% (n = 14,647) of *S. aureus* isolates in this study were methicillin-resistant. MRSA global rates decreased slightly, from 45.0% in 2004 (1,162/2,585) to 39.6% in 2013 (666/1,682); yearly global MRSA levels declined to as low as 30.5% in 2010 (1,319/4,327) (data not shown). MRSA levels exceeded 45% in Asia-Pacific (948/2,061), Latin America (1,904/3,901), and North America (6,733/13,077). Linezolid, vancomycin, tigecycline, and minocycline retained their activity against MRSA isolates (Table 4).

Although overall susceptibility levels were low (<30%), significant increases in levofloxacin susceptibility among MRSA were observed in Europe (p < 0.0001; 11.8% in 2004 and 20.9% in 2012 [10.6% in 2013]), Middle East (p < 0.0001; 17.4% in 2004 and 73.1% in 2013), North America (p < 0.0001; 19.8% in 2004 and 32.7% in 2013), and Latin America (p < 0.01; 3.3% in 2004 and 27.8% in 2012). MRSA susceptibility to minocycline increased significantly in Middle East (p < 0.0001; 30.4% in 2004 and 100% in 2013) but decreased in Asia/Pacific Rim (p < 0.0001; 67.3% in 2004 and 32.4% in 2010 [96.0% in 2012]) and globally (p < 0.01; 97.6% in 2004 and 86.9% in 2010 [98.9% in 2013]) (Additional file 2: Table S2).

#### Streptococcus agalactiae

Globally, 12,819 *S. agalactiae* isolates were collected between 2004 and 2013. Susceptibility was high to most antimicrobial agents with the exception of minocycline, to which only 20.6% of isolates were susceptible (Table 5). MIC_90_s were low for those antimicrobial agents with no *S. agalactiae* breakpoints (amoxicillin-clavulanate, 0.12 mg/L; imipenem, 0.12 mg/L; and piperacillin-tazobactam, 0.5 mg/L).

**Table 5:** **Antimicrobial activity of antimicrobial agents against** ***Streptococcus*** **spp. collected globally between 2004 - 2013**

	**MIC**_**90**_ **(mg/L)**	**MIC range (mg/L)**	**% susceptible**	**% resistant**
***Streptococcus agalactiae*** (n = 12,819)				
AMC	0.12	≤0.03 – ≥ 16	-	-
AMP	0.12	≤0.06 – 0.25	100	-
CRO	0.12	≤0.03 – 0.5	100	-
IPM (n = 2,511)	0.25	≤0.12 – 8	-	-
LVX	1	≤0.06 – ≥ 64	98.6	1.1
LIN	1	≤0.5 – 2	100	-
MEM (n = 10,308)	≤0.12	≤0.12 – 1	99.9	-
MIN	≥16	≤0.25 – ≥ 16	20.6	70.6
PEN	0.12	≤0.06 – 0.12	100	-
TZP	0.5	≤0.25 – ≥ 32	-	-
TGC	0.12	≤0.008 – 2	99.9	-
VAN	0.5	≤0.12 – 1	100	-
***Streptococcus pneumoniae*** (n = 14,562)				
AMC	2	≤0.03 – ≥ 16	92.8	3.5
AMP	4	≤0.06 – ≥ 32	-	-
AZM (n = 12,973)	64	≤0.03 – ≥ 128	67.2	32.4
CRO	1	≤0.03 – ≥ 128	95.0	1.2
CLR (n = 12,973)	64	≤0.015 – ≥ 128	67.5	32.0
CLI (n = 12,973)	≥128	≤0.015 – ≥ 128	80.8	18.9
ERY (n = 12,973)	64	≤0.015 – ≥ 128	66.8	32.7
IPM (n = 3,154)	0.5	≤0.12 – ≥ 32	74.2	4.1
LVX	1	≤0.06 – ≥ 64	98.9	0.7
LIN	1	≤0.5 – 4	99.9	-
MEM (n = 11,408)	0.5	≤0.12 – ≥ 32	81.4	10.0
MIN	8	≤0.25 – ≥ 16	64.4	26.9
PEN	2	≤0.06 – ≥ 16	61.9	14.8
TZP	2	≤0.25 – ≥ 32	-	-
TGC	0.06	≤0.008 – 0.25	99.9	-
VAN	0.5	≤0.12 – 1	100	-
***Streptococcus pneumoniae***, PRSP (n = 2,152)				
AMC	8	≤0.03 – ≥ 16	53.2	22.7
AZM (n = 1,971)	≥128	≤0.03 – ≥ 128	21.5	78.0
CRO	2	0.06 – ≥ 128	69.9	7.2
CLR (n = 1,971)	≥128	≤0.015 – ≥ 128	21.8	77.8
CLI (n = 1,971)	≥128	≤0.015 – ≥ 128	43.2	56.1
ERY (n = 1,971)	≥128	≤0.015 – ≥ 128	20.9	78.7
IPM (n = 357)	1	≤0.12– ≥ 32	1.4	29.7
LVX	1	≤0.06 – ≥ 64	97.5	1.9
LIN	1	≤0.5 – 2	100	-
MEM (n = 1,795)	1	≤0.12 – ≥ 32	6.1	58.4
MIN	≥16	≤0.25 – ≥ 16	27.6	60.3
TZP	8	≤0.25 – ≥ 32	-	-
TGC	0.03	≤0.008 – 0.12	99.8	-
VAN	0.5	≤0.12 – 1	100	-

AMK, amikacin; AMC, amoxicillin-clavulanate; AMP, ampicillin; AZM, azithromycin; FEP, cefepime; CAZ, ceftazidime; CLR, clarithromycin; CLI, clindamycin; CRO, ceftriaxone; ERY, erythromycin; IPM, imipenem; LVX, levofloxacin; LZD, linezolid; MEM, meropenem; MIN, minocycline; PEN, penicillin; PRSP, penicillin-resistant *S. pneumoniae*; TZP, piperacillin-tazobactam; TGC, tigecycline; VAN, vancomycin; VR, vancomycin-resistant.

Statistically significant decreases in susceptibility to levofloxacin (p < 0.001; 100% in 2004 and 74.1% in 2011) and minocycline (p < 0.01; 49.0% in 2005 and 32.0% in 2012) were observed in Asia/Pacific Rim and Latin America, respectively; no statistically significant changes in susceptibility were recorded globally (Additional file 2: Table S2).

#### Streptococcus pneumoniae

Susceptibility among *S. pneumoniae* (n = 14,562) was highest to vancomycin (100%), tigecycline, linezolid (both 99.9%), and levofloxacin (98.9%); susceptibility to ceftriaxone and amoxicillin-clavulanate were also high (95.0% and 92.8%, respectively) (Table 5). Global penicillin susceptibility among *S. pneumoniae* increased significantly during this study (p < 0.0001; 58.0% in 2004 to 64.7% in 2013) but susceptibility decreased to amoxicillin-clavulanate, ceftriaxone, minocycline (each p < 0.0001), and clindamycin (p < 0.001) (Additional file 2: Table S2).

Penicillin-resistant *S. pneumoniae* (PRSP) comprised 14.8% of the *S. pneumoniae* isolates in this study; rates ranged from 11.1% (8/72) in 2013 to 18.0% (350/1,941) in 2008 and percentages were highest in Middle East (24.7%, 114/461), Africa (28.1%, 63/224), and Asia/Pacific Rim (30.1%, 296/985) (data not shown). Vancomycin, tigecycline, linezolid, and levofloxacin retained activity against these resistant isolates; however, susceptibility to the β-lactam and macrolide antimicrobials and minocycline decreased dramatically against PRSP, as much as 75.3% for meropenem (Table 5).

Statistically significant decreases in antimicrobial susceptibility occurred among PRSP in all geographical regions (Additional file 2: Table S2), most notably in North America where reductions were noted for amoxicillin-clavulanate (p < 0.0001; 61.2% in 2004 and 35.2% in 2012), ceftriaxone (p < 0.0001; 89.1% in 2004 and 44.2% in 2009 [77.3% in 2012]), minocycline (p < 0.0001; 50.4% in 2004 and 16.5% in 2011 [27.3% in 2012]), clindamycin (p < 0.001; 55.6% in 2004 and 32.1% in 2012), azithromycin (p < 0.01; 18.3% in 2004 and 7.7% in 2012), clarithromycin (p < 0.01; 19.0% in 2004 and 7.7% in 2012), and erythromycin (p < 0.01; 17.5% in 2004 and 7.7% in 2012). (Additional file 2: Table S2).

## Discussion

Statistically significant changes in antimicrobial susceptibility have been reported among all organisms monitored in this study, with 410 such changes observed here; of these, 331 (81%) represented decreases in susceptibility. Globally, significant changes were observed in 106 cases, 85 of which (80%) denoted decreased susceptibility. Regionally, significant susceptibility changes were most common in Europe (71), Asia/Pacific Rim (67) and North America (68); decreases in susceptibility were reported in 54 (76%), 65 (97%), and 53 (78%) of cases, respectively. Widespread regional declines in antimicrobial susceptibility (or increases in resistance) have previously been shown, including Europe [11], Asia [12], and North America [13].

Statistically significant changes in susceptibility were observed most often among *A. baumannii* (32), *E. coli* (34), *K. pneumoniae* (30), and *S. pneumoniae* (31); among these, significant decreases in susceptibility were observed in 30 (94%), 33 (97%), 29 (97%) and 29 (94%) cases, respectively. These changes reflect other recent reports of declining susceptibility among important pathogens, including *P. aeruginosa* [13], *E. coli* [14], *K. pneumoniae* [15,16], and *S. pneumoniae* [17].

*A. baumannii* susceptibility decreased in this report, both globally and regionally, to most antimicrobials; 47.1% of *A. baumannii* isolates from Asia/Pacific Rim were MDR. Molton et al. [12] reported similar results, with 55% of *A. baumannii* isolates from Singapore being MDR. *A. baumannii* are adept at acquiring resistance mechanisms and in expanding their global distribution [18]; for example, meropenem susceptibility among *A. baumannii* from a tertiary care teaching hospital in Mexico decreased from 91.7% in 1999 to 11.8% in 2011 while imipenem susceptibility decreased from 88.2% to 13.9% [19]. These examples highlight the importance of monitoring highly resistant pathogens such as *A. baumannii*, which have the potential to become pan-resistant [12].

The activity of tigecycline against resistant clinical bacteria collected globally in 2011 was described by Sader et al. [20] as a part of the SENTRY Antimicrobial Surveillance Program. Tigecycline susceptibility levels of 100% were reported among MRSA, 99.9% among ESBL-positive *E. coli*, 99.5% among vancomycin-resistant *Enterococcus* spp., 99.4% among PRSP, and 97.7% among ESBL-positive *Klebsiella* spp.; also, a tigecycline MIC_90_ of 2 mg/L was reported by Sader et al. [20] for *Acinetobacter*. In a separate study, Sader et al. [21] reported good tigecycline activity against clinical isolates collected in the USA between 2006 and 2012, including MDR phenotypes, with no upwards trends in tigecycline resistance reported. These results accentuate the good in vitro activity of tigecycline against resistance phenotypes in the current study, including ESBL-positive and carbapenem-resistant *E. coli*, vancomycin-resistant enterococci, MRSA, and PRSP. Tigecycline was also active against carbapenem-resistant *Enterobacter* spp., with 95.4% of all isolates and 83.0% of carbapenem-resistant isolates susceptible to tigecycline; the next most active antimicrobial against carbapenem-resistant isolates was amikacin, with 68.3% of isolates susceptible. The good in vitro activity of tigecycline against resistant pathogens suggests it may have an important role in the treatment of infections caused by these difficult-to-treat pathogens.

Global susceptibility of *Enterobacter* spp., *K. pneumoniae*, *S. marcescens*, and *S. pneumoniae* to minocycline increased in this study by ≥20% between 2011 and 2012; this increase has not been reported in previously published studies. The numbers of centres participating in the TEST study increased globally from 197 in 2011 to 398 in 2012; this large influx of isolates from new centres in 2012 may be responsible for the observed susceptibility changes that year. This highlights one of the limitations of longitudinal surveillance studies: inconsistent centre involvement over time, with some centres contributing isolates in several years but others in only a single year. Another bias in TEST is the regional distribution of centres: Europe and the US account for more than two thirds of the centres participating in this study (72.9%, 443/608), thus global results reported are heavily influenced by trends in these regions.

Antimicrobial surveillance monitors the epidemiology of and changes in antimicrobial resistance as well as helping to reduce the spread of resistant organisms. Longitudinal surveillance studies such as the Study for Monitoring Antimicrobial Resistance Trends (SMART), SENTRY, and TEST are thus important tools in the development of guidelines for rational empiric antimicrobial therapy and, more immediately, directing local empiric therapy [5,20,22,23]. The development and rapid distribution of carbapenemases such as New Delhi metallo-β-lactamase in recent years highlights the importance of multinational and global antimicrobial surveillance [24,25].

## Conclusions

Antimicrobial susceptibility decreased both globally and regionally between 2004 and 2013 in TEST among clinically important pathogens such as *A. baumannii* and *E. coli.* These results mirror decreases in antimicrobial susceptibility shown in other surveillance studies, such as SENTRY [20] and EARS-Net [11]. The sustained high levels of susceptibility to tigecycline among most of the pathogens examined in this study, including multidrug-resistant pathogens such as ESBL-positive *E. coli* and penicillin-resistant *S. pneumoniae*, suggest that tigecycline may continue to be useful in the treatment of infectious diseases in coming years.

## Additional files

Additional file 1: Table S1. Statistically significant (p < 0.01) changes in antimicrobial susceptibility between 2004–2013 among gram-negative pathogens. Additional file 2: Table S2. Statistically significant (p < 0.01) changes in antimicrobial susceptibility between 2004–2013 among gram-positive pathogens.
