# Supplementary material for: Global in vitro activity of tigecycline and comparator agents: Tigecycline Evaluation and Surveillance Trial 2004–2013
Source: Ann Clin Microbiol Antimicrob. 2015 May 10;14:27. doi: 10.1186/s12941-015-0085-1 (PMC4489028; doi:10.1186/s12941-015-0085-1)
Supplement: Additional file 1: Table S1. — Statistically significant (p < 0.01) changes in antimicrobial susceptibility between 2004–2013 among gram-negative pathogens. [file 12941_2015_85_MOESM1_ESM.doc]

## Additional file 1: Table S1. Statistically significant (p<0.01) changes in antimicrobial susceptibility between 2004 - 2013 among gram-negative pathogens

|  |  | **2004**–**2013** | | **Susceptibility Change  2004**–**2013^b^** |
| --- | --- | --- | --- | --- |
|  |  | **MIC_90_  (mg/L)** | **% Susceptible** |  |
| **Enterobacteriaceae** |  |  |  |  |
| Africa |  | n=2087 | |  |
|  | FEP | ≥64 | 78.6 | p<0.0001 |
|  | CRO | 64 | 63.2 | p<0.0001 |
|  | LVX | ≥16 | 77.1 | p<0.0001 |
| Asia/Pacific Rim |  | n=7191 | |  |
|  | AMK | 8 | 94.0 | p<0.0001 |
|  | FEP | ≥64 | 82.2 | p<0.0001 |
|  | CRO | ≥128 | 60.9 | p<0.0001 |
|  | LVX | ≥16 | 73.8 | p<0.0001 |
|  | MIN | 16 | 70.0 | p<0.0001 |
|  | TZP | 64 | 81.7 | p<0.0001 |
|  | TGC | 1 | 96.8 | p<0.0001 |
| Europe |  | n=51,074 | |  |
|  | AMC | ≥64 | 42.0 | p<0.01 |
|  | FEP | 16 | 88.2 | p<0.0001 |
|  | CRO | 64 | 70.5 | p<0.0001 |
|  | LVX | ≥16 | 79.0 | p<0.0001 |
|  | MIN | 16 | 73.3 | p<0.001 |
| Latin America |  | n=12,752 | |  |
|  | CRO | ≥128 | 56.3 | p<0.01 |
|  | LVX | ≥16 | 65.0 | p<0.0001 |
|  | MIN | ≥32 | 60.0 | p<0.0001 |
|  | TGC | 2 | 97.0 | p<0.01 |
| Middle East |  | n=4004 | |  |
|  | TGC | 2 | 96.0 | p<0.01 |
| North America |  | n=41,791 | |  |
|  | FEP | 2 | 95.6 | p<0.0001 |
|  | CRO | 16 | 82.9 | p<0.0001 |
|  | LVX | 8 | 85.1 | p<0.0001 |
|  | MIN | 8 | 83.2 | p<0.0001 |
|  | TZP | 16 | 90.6 | p<0.0001 |
|  | TGC | 1 | 96.9 | p<0.01 (+) |
| Global |  | n=118,899 (118,648)^a^ | |  |
|  | AMC | ≥64 | 43.5 | p<0.0001 |
|  | AMP | ≥64 | 13.0 | p<0.001 |
|  | FEP | 16 | 88.7 | p<0.0001 |
|  | CRO | 64 | 72.3 | p<0.0001 |
|  | LVX | ≥16 | 79.0 | p<0.0001 |
|  | MIN | 16 | 74.9 | p<0.0001 |
|  | TZP | 64 | 84.0 | p<0.0001 |
| **Enterobacteriaceae**, MDR |  |  |  |  |
| Africa |  | n=187 | |  |
|  | FEP | ≥64 | 31.6 | p<0.001 |
|  | MIN | ≥32 | 11.8 | p<0.001 (+) |
| Asia/Pacific Rim |  | n=818 | |  |
|  | AMK | ≥128 | 61.2 | p<0.001 |
|  | FEP | ≥64 | 40.7 | p<0.01 |
| Europe |  | n=3,987 | |  |
|  | AMP | ≥64 | 0.3 | p<0.01 |
|  | FEP | ≥64 | 47.7 | p<0.0001 |
|  | CRO | ≥128 | 20.7 | p<0.01 |
|  | MIN | ≥32 | 10.5 | p<0.0001 (+) |
| Latin America |  | n=2,084 | |  |
|  | AMK | ≥128 | 61.9 | p<0.0001 (+) |
|  | FEP | ≥64 | 42.7 | p<0.01 (+) |
|  | LVX | ≥16 | 5.6 | p<0.01 |
|  | MIN | ≥32 | 11.6 | p<0.001 |
| North America |  | n=1,801 | |  |
|  | FEP | ≥64 | 59.2 | p<0.0001 |
|  | CRO | ≥128 | 27.2 | p<0.01 |
|  | TGC | 8 | 73.9 | p<0.0001 (+) |
| Global |  | n=9,372 | |  |
|  | AMK | ≥128 | 75.4 | p<0.01 (+) |
|  | FEP | ≥64 | 47.3 | p<0.0001 |
|  | CRO | ≥128 | 20.3 | p<0.0001 |
|  | LVX | ≥16 | 6.1 | p<0.0001 |
|  | MIN | ≥32 | 12.0 | p<0.01 (+) |
|  | TZP | ≥256 | 39.5 | p<0.0001 |
|  | TGC | 4 | 83.2 | p<0.001 (+) |
| ***Enterobacter* spp.** |  |  |  |  |
| Africa |  | n=553 | |  |
|  | FEP | 32 | 81.9 | p<0.0001 |
|  | CRO | 64 | 60.9 | p<0.0001 |
| Asia/Pacific Rim |  | n= 2,025 | |  |
|  | AMK | 8 | 94.7 | p<0.0001 |
|  | FEP | 32 | 86.2 | p<0.001 |
|  | CRO | ≥128 | 51.1 | p<0.01 |
|  | LVX | 8 | 85.7 | p<0.001 |
|  | MIN | 16 | 71.1 | p<0.0001 |
|  | TZP | 128 | 71.2 | p<0.01 |
| Europe |  | n=14,476 | |  |
|  | FEP | 8 | 91.4 | p<0.0001 |
|  | LVX | 8 | 84.4 | p<0.01 (+) |
|  | MIN | 16 | 71.2 | p<0.01 |
|  | TZP | 128 | 72.3 | p<0.01 (+) |
| Latin America |  | n=3,304 | |  |
|  | FEP | ≥64 | 81.9 | p<0.0001 (+) |
|  | MIN | ≥32 | 60.6 | p<0.0001 |
|  | TZP | ≥256 | 70.9 | p<0.01 (+) |
|  | TGC | 2 | 95.6 | p<0.01 |
| North America |  | n=11,534 (11,468)^a^ | |  |
|  | LVX | 1 | 92.3 | p<0.001 (+) |
|  | MIN | 8 | 81.7 | p<0.0001 |
|  | TGC | 2 | 95.0 | p<0.0001 (+) |
| Global |  | n=32,987 (32,857)^a^ | |  |
|  | FEP | 8 | 91.7 | p<0.0001 |
|  | CRO | 64 | 61.7 | p<0.0001 |
|  | MIN | 16 | 73.7 | p<0.0001 |
|  | TZP | 128 | 76.1 | p<0.0001 |
|  | TGC | 2 | 95.4 | p<0.001 (+) |
| ***Enterobacter* spp.**, Car-R | |  |  |  |
| Europe |  | n=269 | |  |
|  | CRO | ≥128 | 5.2 | p<0.01 |
|  | LVX | ≥16 | 37.9 | p<0.01 |
|  | TZP | ≥256 | 19.7 | p<0.0001 |
| Global |  | n=578 | |  |
|  | CRO | ≥128 | 6.7 | p<0.001 |
|  | MIN | ≥32 | 39.8 | p<0.01 |
|  | TZP | ≥256 | 22.5 | p<0.01 |
| ***Escherichia coli*** |  |  |  |  |
| Africa |  | n=661 | |  |
|  | FEP | 32 | 86.2 | p<0.0001 |
|  | CRO | 64 | 76.6 | p<0.0001 |
|  | LVX | ≥16 | 69.6 | p<0.0001 |
| Asia/Pacific Rim |  | n=2,177 | |  |
|  | AMK | 8 | 95.6 | p<0.0001 |
|  | FEP | ≥64 | 78.8 | p<0.001 |
|  | CRO | ≥128 | 63.7 | p<0.0001 |
|  | LVX | ≥16 | 55.3 | p<0.0001 |
|  | MIN | 16 | 67.8 | p<0.0001 |
|  | TZP | 32 | 89.5 | p<0.01 |
| Europe |  | n=15,754 | |  |
|  | AMP | ≥64 | 34.4 | p<0.0001 |
|  | FEP | 16 | 87.8 | p<0.0001 |
|  | CRO | 64 | 78.4 | p<0.0001 |
|  | LVX | ≥16 | 68.4 | p<0.0001 |
| Latin America |  | n=4,239 | |  |
|  | AMC | 32 | 51.1 | p<0.001 |
|  | AMP | ≥64 | 21.3 | p<0.0001 |
|  | CRO | ≥128 | 61.2 | p<0.0001 |
|  | LVX | ≥16 | 47.7 | p<0.0001 |
|  | MIN | ≥32 | 59.3 | p<0.0001 |
|  | TZP | 32 | 85.1 | p<0.001 |
| Middle East |  | n=1,273 | |  |
|  | AMC | 32 | 53.1 | p<0.0001 (+) |
| North America |  | n=12,934 | |  |
|  | AMC | 32 | 74.7 | p<0.01 |
|  | AMP | ≥64 | 45.4 | p<0.0001 |
|  | FEP | 1 | 95.7 | p<0.0001 |
|  | CRO | 1 | 90.2 | p<0.0001 |
|  | TZP | 8 | 95.2 | p<0.0001 |
|  | TGC | 0.25 | 99.9 | p<0.01 |
| Global |  | n=37,038 | |  |
|  | AMC | 32 | 66.6 | p<0.0001 |
|  | AMP | ≥64 | 35.6 | p<0.0001 |
|  | FEP | 16 | 88.5 | p<0.0001 |
|  | CRO | 64 | 79.1 | p<0.0001 |
|  | LVX | ≥16 | 66.1 | p<0.0001 |
|  | MIN | 16 | 77.0 | p<0.0001 |
|  | TZP | 16 | 91.3 | p<0.0001 |
|  | TGC | 0.5 | 99.9 | p<0.01 |
| ***Escherichia coli***, ESBL-positive | |  | |  |
| Asia/Pacific Rim |  | n=469 | |  |
|  | AMC | 32 | 37.5 | p<0.0001 (+) |
|  | MIN | ≥32 | 59.3 | p<0.01 |
| Europe |  | n=2,590 | |  |
|  | AMK | 16 | 95.3 | p<0.0001 (+) |
|  | AMC | 32 | 33.5 | p<0.0001 (+) |
|  | AMP | ≥64 | 0.3 | p<0.01 |
|  | CRO | ≥128 | 1.0 | p<0.001 |
|  | MIN | 16 | 67.4 | p<0.0001 (+) |
|  | TZP | 128 | 75.8 | p<0.01 (+) |
| Latin America |  | n=1,049 | |  |
|  | MIN | ≥32 | 51.6 | p<0.001 |
|  | TZP | 128 | 72.4 | p<0.001 |
| Middle East |  | n=330 | |  |
|  | AMC | ≥64 | 28.8 | p<0.0001 (+) |
|  | MIN | ≥32 | 58.8 | p<0.01 (+) |
| North America |  | n=650 | |  |
|  | AMC | 32 | 34.8 | p<0.01 (+) |
|  | FEP | ≥64 | 32.0 | p<0.0001 |
|  | CRO | ≥128 | 2.8 | p<0.0001 |
|  | MIN | 16 | 70.3 | p<0.001 (+) |
| Global |  | n=5,178 | |  |
|  | AMK | 16 | 94.2 | p<0.0001 (+) |
|  | AMC | 32 | 31.3 | p<0.0001 (+) |
|  | AMP | ≥64 | 0.4 | p<0.01 |
|  | FEP | ≥64 | 32.4 | p<0.01 |
|  | CRO | ≥128 | 1.3 | p<0.0001 |
|  | LVX | ≥16 | 18.1 | p<0.01 (+) |
|  | MIN | ≥32 | 63.2 | p<0.0001 (+) |
| ***Escherichia coli***, Car-R |  |  |  |  |
| Global |  | n=181 | |  |
|  | FEP | ≥64 | 28.2 | p<0.0001 |
|  | CRO | ≥128 | 9.9 | p<0.01 |
|  | TGC | 2 | 97.2 | p<0.01 |
| ***Klebsiella oxytoca*** |  |  |  |  |
| Asia/Pacific Rim |  | n=245 | |  |
|  | AMK | 4 | 94.7 | p<0.0001 |
|  | LVX | 4 | 87.8 | p<0.01 |
|  | MIN | 8 | 84.5 | p<0.0001 |
| Europe |  | n=3,887 | |  |
|  | TGC | 1 | 98.8 | p<0.01 (+) |
| Latin America |  | n=365 | |  |
|  | LVX | ≥16 | 80.8 | p<0.01 |
|  | MIN | 16 | 77.8 | p<0.01 |
| Global |  | n=6,940 | |  |
|  | TZP | ≥256 | 85.1 | p<0.01 |
|  | TGC | 1 | 98.7 | p<0.01 (+) |
| ***Klebsiella pneumoniae*** |  |  |  |  |
| Africa |  | n=582 | |  |
|  | FEP | ≥64 | 59.3 | p<0.01 |
|  | LVX | ≥16 | 67.0 | p<0.01 |
| Asia/Pacific Rim |  | n=1,937 | |  |
|  | AMK | 16 | 90.9 | p<0.001 |
|  | FEP | ≥64 | 76.6 | p<0.001 |
|  | CRO | ≥128 | 61.3 | p<0.01 |
|  | LVX | ≥16 | 73.6 | p<0.0001 |
|  | MIN | ≥32 | 70.3 | p<0.0001 |
|  | TZP | ≥256 | 79.6 | p<0.0001 |
|  | TGC | 2 | 95.5 | p<0.01 |
| Europe |  | n=11,427 | |  |
|  | AMK | 8 | 95.3 | p<0.0001 |
|  | AMC | 32 | 63.3 | p<0.0001 |
|  | FEP | ≥64 | 77.8 | p<0.0001 |
|  | CRO | ≥128 | 67.5 | p<0.0001 |
|  | LVX | ≥16 | 75.1 | p<0.0001 |
|  | MIN | ≥32 | 68.5 | p<0.01 |
|  | TZP | ≥256 | 76.6 | p<0.0001 |
| Latin America |  | n=3,501 | |  |
|  | MIN | ≥32 | 58.6 | p<0.0001 |
| Middle East |  | n=1,115 | |  |
|  | TZP | ≥256 | 66.2 | p<0.001 (+) |
|  | TGC | 2 | 93.0 | p<0.01 |
| North America |  | n=10,366 | |  |
|  | FEP | 4 | 93.0 | p<0.0001 |
|  | CRO | 16 | 84.8 | p<0.01 |
|  | LVX | 8 | 86.6 | p<0.01 |
|  | MIN | 16 | 80.6 | p<0.0001 |
| Global |  | n=28,928 | |  |
|  | AMK | 16 | 94.4 | p<0.0001 |
|  | AMC | 32 | 67.9 | p<0.0001 |
|  | FEP | ≥64 | 81.0 | p<0.0001 |
|  | CRO | ≥128 | 70.1 | p<0.0001 |
|  | LVX | ≥16 | 77.4 | p<0.0001 |
|  | MIN | 16 | 71.4 | p<0.0001 |
|  | TZP | ≥256 | 79.4 | p<0.0001 |
| ***Klebsiella pneumoniae***, ESBL-positive | | |  |  |
| Africa |  | n=249 | |  |
|  | FEP | ≥64 | 28.1 | p<0.01 |
| Asia/Pacific Rim |  | n=462 | |  |
|  | MIN | ≥32 | 47.0 | p<0.0001 |
| Europe |  | n=2,593 | |  |
|  | AMK | 32 | 89.1 | p<0.01 (+) |
|  | FEP | ≥64 | 26.5 | p<0.0001 |
|  | LVX | ≥16 | 34.1 | p<0.001 |
|  | MIN | ≥32 | 45.2 | p<0.0001 (+) |
|  | TZP | ≥256 | 45.3 | p<0.01 (+) |
| Latin America |  | n=1,275 | |  |
|  | MIN | ≥32 | 47.1 | p<0.0001 |
| Middle East |  | n=337 | |  |
|  | TZP | ≥256 | 49.0 | p<0.001 (+) |
| North America |  | n=983 | |  |
|  | FEP | ≥64 | 49.2 | p<0.01 |
|  | MIN | ≥32 | 57.1 | p<0.001 |
| Global |  | n=5,899 | |  |
|  | AMK | 32 | 83.6 | p<0.0001 (+) |
|  | FEP | ≥64 | 31.9 | p<0.0001 |
|  | CRO | ≥128 | 1.4 | p<0.001 |
|  | MIN | ≥32 | 48.4 | p<0.0001 |
|  | TZP | ≥256 | 44.3 | p<0.01 (+) |
| ***Klebsiella pneumoniae***, Car-R | |  |  |  |
| Africa |  | n=45 | |  |
|  | MIN | ≥32 | 35.6 | p<0.0001 (+) |
| Europe |  | n=540 | |  |
|  | FEP | ≥64 | 4.4 | p<0.01 |
|  | LVX | ≥16 | 8.7 | p<0.001 |
|  | TZP | ≥256 | 2.8 | p<0.01 |
| Latin America |  | n=209 | |  |
|  | LVX | ≥16 | 11.5 | p<0.001 |
|  | TZP | ≥256 | 8.1 | p<0.01 |
| Global |  | n=1,330 | |  |
|  | AMC | ≥64 | 1.7 | p<0.001 |
|  | FEP | ≥64 | 7.4 | p<0.0001 |
|  | CRO | ≥128 | 1.8 | p<0.0001 |
|  | LVX | ≥16 | 10.1 | p<0.0001 |
|  | TZP | ≥256 | 4.7 | p<0.0001 |
| ***Serratia marcescens*** |  |  |  |  |
| Asia/Pacific Rim |  | n=807 | |  |
|  | MIN | 16 | 67.9 | p<0.0001 |
|  | TGC | 2 | 93.8 | p<0.001 |
| Europe |  | n=5,530 (5,515)^a^ | |  |
|  | AMC | ≥64 | 3.5 | p<0.001 (+) |
|  | AMP | ≥64 | 3.6 | p<0.001 (+) |
|  | MIN | 8 | 67.9 | p<0.0001 |
|  | TZP | 16 | 92.4 | p<0.01 (+) |
| Latin America |  | n=1,343 | |  |
|  | MIN | 16 | 60.0 | p<0.0001 |
|  | TGC | 2 | 94.9 | p<0.01 |
| Middle East |  | n=415 | |  |
|  | FEP | 4 | 93.0 | p<0.01 (+) |
| North America |  | n=4,697 (4,684)^a^ | |  |
|  | AMP | ≥64 | 2.0 | p<0.001 (+) |
|  | LVX | 1 | 94.7 | p<0.01 |
|  | MIN | 8 | 82.4 | p<0.0001 |
|  | TZP | 8 | 95.6 | p<0.01 |
| Global |  | n=13,006 (12,963)^a^ | |  |
|  | AMC | ≥64 | 3.3 | p<0.01 (+) |
|  | AMP | ≥64 | 2.7 | p<0.0001 (+) |
|  | MIN | 8 | 72.3 | p<0.0001 |
| ***Serratia marcescens***, Car-R | |  |  |  |
| Global |  | n=229 | |  |
|  | LVX | ≥16 | 60.7 | p<0.01 |
| ***Acinetobacter baumannii*** |  |  |  |  |
| Africa |  | n=340 | |  |
|  | FEP | ≥64 | 19.4 | p<0.0001 |
|  | CAZ | ≥64 | 20.0 | p<0.01 |
|  | CRO | ≥128 | 11.2 | p<0.0001 |
|  | LVX | ≥16 | 26.5 | p<0.0001 |
|  | MIN | 16 | 63.2 | p<0.01 (+) |
|  | TZP | ≥256 | 21.2 | p<0.0001 |
| Asia/Pacific Rim |  | n=1,228 | |  |
|  | AMK | ≥128 | 52.4 | p<0.0001 |
|  | FEP | ≥64 | 39.7 | p<0.0001 |
|  | CAZ | ≥64 | 38.3 | p<0.001 |
|  | CRO | ≥128 | 19.2 | p<0.0001 |
|  | LVX | ≥16 | 44.4 | p<0.0001 |
|  | MIN | 8 | 80.1 | p<0.0001 |
|  | TZP | ≥256 | 38.6 | p<0.0001 |
| Europe |  | n=7,082 | |  |
|  | AMK | ≥128 | 62.5 | p<0.0001 |
|  | FEP | ≥64 | 50.4 | p<0.0001 |
|  | CAZ | ≥64 | 45.9 | p<0.0001 |
|  | CRO | ≥128 | 25.8 | p<0.0001 |
|  | LVX | ≥16 | 47.4 | p<0.0001 |
|  | MIN | 8 | 84.7 | p<0.0001 |
|  | TZP | ≥256 | 45.4 | p<0.0001 |
| Latin America |  | n=2,048 | |  |
|  | MIN | 8 | 89.4 | p<0.0001 |
| Middle East |  | n=673 | |  |
|  | MIN | 8 | 68.4 | p<0.0001 |
| North America |  | n=5,407 | |  |
|  | AMK | ≥128 | 78.2 | p<0.0001 |
|  | FEP | ≥64 | 49.7 | p<0.01 (+) |
|  | TZP | ≥256 | 55.3 | p<0.01 |
| Global |  | n=16,778 | |  |
|  | AMK | ≥128 | 61.1 | p<0.0001 |
|  | FEP | ≥64 | 44.3 | p<0.0001 |
|  | CAZ | ≥64 | 41.2 | p<0.0001 |
|  | CRO | ≥128 | 23.3 | p<0.0001 |
|  | LVX | ≥16 | 43.0 | p<0.0001 |
|  | MIN | 8 | 84.5 | p<0.0001 |
|  | TZP | ≥256 | 42.9 | p<0.0001 |
| ***Acinetobacter baumannii***, MDR | |  |  |  |
| Africa |  | n=202 | |  |
|  | MIN | 16 | 49.0 | p<0.001 (+) |
| Asia/Pacific Rim |  | n=579 | |  |
|  | LVX | ≥16 | 4.7 | p<0.01 |
|  | MIN | 16 | 62.7 | p<0.0001 |
| Europe |  | n=2,616 | |  |
|  | FEP | ≥64 | 7.3 | p<0.01 |
|  | MIN | 16 | 67.0 | p<0.0001 |
|  | TZP | ≥256 | 2.4 | p<0.001 |
| Latin America |  | n=1,388 | |  |
|  | AMK | ≥128 | 8.8 | p<0.001 (+) |
|  | MIN | 8 | 87.8 | p<0.0001 |
| Middle East |  | n=452 | |  |
|  | MIN | 16 | 57.7 | p<0.001 |
| North America |  | n=1,506 | |  |
|  | TZP | ≥256 | 5.5 | p<0.001 |
| Global |  | n=6,743 | |  |
|  | MIN | 16 | 70.3 | p<0.0001 |
|  | TZP | ≥256 | 3.0 | p<0.0001 |
| ***Haemophilus influenzae*** |  |  |  |  |
| Africa |  | n=269 | |  |
|  | FEP | ≤0.5 | 98.9 | p<0.01 |
| Asia/Pacific Rim |  | n=990 | |  |
|  | TGC | 0.25 | 98.1 | p<0.001 |
| Europe |  | n=7,040 | |  |
|  | MIN | 1 | 98.3 | p<0.0001 (+) |
| Latin America |  | n=1,157 | |  |
|  | CRO | ≤0.06 | 99.7 | p<0.01 |
|  | TZP | ≤0.06 | 99.2 | p<0.01 |
|  | TGC | 0.25 | 97.6 | p<0.01 |
| Global |  | n=15,925 | |  |
|  | AMP | 32 | 78.3 | p<0.001 (+) |
|  | CRO | ≤0.06 | 99.9 | p<0.0001 |
|  | TGC | 0.25 | 98.9 | p<0.0001 |
| ***Pseudomonas aeruginosa*** |  |  |  |  |
| Asia/Pacific Rim |  | n=1,769 | |  |
|  | FEP | ≥64 | 67.6 | p<0.01 |
|  | CAZ | ≥64 | 64.8 | p<0.0001 |
|  | TZP | ≥256 | 66.1 | p<0.0001 |
| Europe |  | n=12,050 | |  |
|  | LVX | ≥16 | 64.9 | p<0.01 (+) |
| Latin America |  | n=3,077 | |  |
|  | CAZ | ≥64 | 54.5 | p<0.01 |
|  | TZP | ≥256 | 56.4 | p<0.01 |
| Middle East |  | n=950 | |  |
|  | TZP | 128 | 67.7 | p<0.01 (+) |
| North America |  | n=10,074 | |  |
|  | LVX | ≥16 | 66.0 | p<0.0001 (+) |
| Global |  | n=28,413 | |  |
|  | AMK | 16 | 90.2 | p<0.01 |
|  | CAZ | 32 | 74.0 | p<0.01 |
|  | LVX | ≥16 | 63.9 | p<0.0001 (+) |
|  | TZP | 128 | 73.1 | p<0.0001 |
| ***Pseudomonas aeruginosa***, MDR | |  |  |  |
| Asia/Pacific Rim |  | n=324 | |  |
|  | AMK | ≥128 | 32.1 | p<0.01 (+) |
| Latin America |  | n=854 | |  |
|  | FEP | ≥64 | 9.5 | p<0.01 (+) |
|  | LVX | ≥16 | 2.2 | p<0.01 (+) |
| North America |  | n=666 | |  |
|  | TZP | ≥256 | 10.8 | p<0.01 |
| Global |  | n=3,496 | |  |
|  | CAZ | ≥64 | 11.7 | p<0.01 |
|  | TZP | ≥256 | 10.9 | p<0.01 |

Data are not presented where n<20 (*H. influenzae*, BL-pos: Africa, n=13 [2004–07], n=15 [2008–13]. *Enterobacter* spp., Car-R: Africa, n=11 [2004–07], n=7 [2008–13]. *E. coli*, Car-R: Africa, n=8 [2004–07], n=5 [2008–13]; Asia/Pacific Rim, n=4 [2004–07], n=10 [2008–13]; Middle East, n=7 [2004–07], n=8 [2008–13]. *Serratia marcescens*, Car-R: Africa, n=1 [2004–07], n=3 [2008–13]; Asia/Pacific Rim, n=8 [2004–07], n=6 [2008–13]; Middle East, n=2 [2004–07], n=5 [2008–13].). Imipenem significance results are not presented where n<50.

Car-R, carbapenem-resistant; ESBL, extended-spectrum β-lactamase; MDR, multidrug-resistant.

^a^ The n values in parentheses represent the numbers of isolates tested against ampicillin.

^b^ Changes in significance indicated by (+) represent increased susceptibility; all others represent decreased susceptibility. A cut-off of p<0.01 was used for statistical significance testing.
