# Supplementary material for: Global in vitro activity of tigecycline and comparator agents: Tigecycline Evaluation and Surveillance Trial 2004–2013
Source: Ann Clin Microbiol Antimicrob. 2015 May 10;14:27. doi: 10.1186/s12941-015-0085-1 (PMC4489028; doi:10.1186/s12941-015-0085-1)
Supplement: Additional file 2: Table S2. — Statistically significant (p < 0.01) changes in antimicrobial susceptibility between 2004–2013 among gram-positive pathogens. [file 12941_2015_85_MOESM2_ESM.doc]

## Additional file 2: Table S2. Statistically significant (p<0.01) changes in antimicrobial susceptibility between 2004 - 2013 among gram-positive pathogens

|  |  | **2004**–**2013** | | **Susceptibility Change  2004**–**2013^b^** |
| --- | --- | --- | --- | --- |
|  |  | **MIC_90_  (mg/L)** | **% Susceptible** |  |
| ***Enterococcus faecalis*** |  |  |  |  |
| Europe |  | n=6,045 | |  |
|  | AMP | 2 | 99.2 | p<0.0001 |
|  | MIN | ≥16 | 30.7 | p<0.001 |
|  | PEN | 4 | 99.0 | p<0.0001 |
| Latin America |  | n=1,700 | |  |
|  | LVX | ≥64 | 68.2 | p<0.01 |
|  | MIN | ≥16 | 34.9 | p<0.001 |
|  | VAN | 2 | 98.2 | p<0.001 (+) |
| North America |  | n=5,319 | |  |
|  | AMP | 1 | 99.7 | p<0.0001 |
|  | LVX | ≥64 | 60.8 | p<0.0001 (+) |
|  | LIN | 2 | 99.7 | p<0.01 (+) |
|  | MIN | ≥16 | 41.8 | p<0.0001 |
|  | PEN | 4 | 99.7 | p<0.0001 |
| Global |  | n=14,615 | |  |
|  | AMP | 2 | 99.4 | p<0.0001 |
|  | LVX | ≥64 | 64.9 | p<0.0001 (+) |
|  | MIN | ≥16 | 34.8 | p<0.0001 |
|  | PEN | 4 | 99.2 | p<0.0001 |
|  | VAN | 2 | 97.5 | p<0.01 (+) |
| ***Enterococcus faecalis***, VR |  |  | |  |
| North America |  | n=230 | |  |
|  | AMP | 2 | 94.8 | p<0.0001 |
|  | MIN | ≥16 | 49.1 | p<0.0001 |
|  | PEN | 8 | 95.2 | p<0.0001 |
| Global |  | n=325 | |  |
|  | AMP | 4 | 94.5 | p<0.0001 |
|  | MIN | ≥16 | 44.3 | p<0.0001 |
|  | PEN | 8 | 94.5 | p<0.001 |
| ***Enterococcus faecium*** |  |  |  |  |
| Asia/Pacific Rim |  | n=433 | |  |
|  | MIN | ≥16 | 66.1 | p<0.0001 |
|  | PEN | ≥16 | 12.0 | p<0.01 |
| Europe |  | n=2,844 | |  |
|  | AMP | ≥32 | 15.2 | p<0.01 |
|  | LVX | ≥64 | 13.8 | p<0.0001 |
| Latin America |  | n=498 | |  |
|  | VAN | ≥64 | 57.8 | p<0.01 (+) |
| Middle East |  | n=189 | |  |
|  | VAN | ≥64 | 72.5 | p<0.01 |
| North America |  | n=2,152 | |  |
|  | MIN | ≥16 | 65.9 | p<0.0001 |
|  | TGC | 0.12 | 99.5 | p<0.01 |
| Global |  | n=6,167 | |  |
|  | MIN | ≥16 | 68.5 | p<0.0001 |
|  | VAN | ≥64 | 64.0 | p<0.0001 (+) |
| ***Enterococcus faecium***, VR |  |  |  |  |
| Asia/Pacific Rim |  | n=92 | |  |
|  | MIN | ≥16 | 70.7 | p<0.001 |
| Latin America |  | n=196 | |  |
|  | AMP | ≥32 | 1.0 | p<0.01 |
| North America |  | n=1,438 | |  |
|  | MIN | ≥16 | 62.9 | p<0.0001 |
|  | TGC | 0.12 | 99.2 | p<0.01 |
| Global |  | n=2,136 | |  |
|  | MIN | ≥16 | 65.0 | p<0.0001 |
| ***Staphylococcus aureus*** |  |  |  |  |
| Asia/Pacific Rim |  | n=2,061 | |  |
|  | LVX | 32 | 58.3 | p<0.0001 |
|  | MIN | 8 | 82.2 | p<0.0001 |
| Europe |  | n=15,551 | |  |
|  | LVX | 16 | 70.9 | p<0.0001 |
| Latin America |  | n=3,910 | |  |
|  | MIN | 1 | 97.5 | p<0.0001 |
| Middle East |  | n=1,228 | |  |
|  | MIN | 1 | 95.0 | p<0.0001 (+) |
| North America |  | n=13,077 | |  |
|  | LVX | 32 | 57.9 | p<0.0001 (+) |
|  | PEN | ≥16 | 9.5 | p<0.0001 (+) |
| Global |  | n=36,448 | |  |
|  | LVX | 32 | 64.1 | p<0.0001 (+) |
|  | LIN | 4 | 99.9 | p<0.01 |
|  | MIN | 0.5 | 97.3 | p<0.01 |
|  | PEN | ≥16 | 10.9 | p<0.0001 (+) |
| ***Staphylococcus aureus***, MRSA | |  | |  |
| Asia/Pacific Rim |  | n=948 ^a^ | |  |
|  | MIN | ≥16 | 63.4 | p<0.0001 |
| Europe |  | n=4,482 | |  |
|  | LVX | 32 | 14.8 | p<0.0001 (+) |
| Latin America |  | n=1,904 | |  |
|  | LVX | 32 | 17.8 | p<0.01 (+) |
| Middle East |  | n=401 | |  |
|  | LVX | 32 | 28.9 | p<0.0001 (+) |
|  | MIN | 8 | 86.5 | p<0.0001 (+) |
| North America |  | n=6,733 | |  |
|  | LVX | ≥64 | 28.2 | p<0.0001 (+) |
| Global |  | n=14,647 | |  |
|  | MIN | 2 | 94.7 | p<0.01 |
| ***Streptococcus agalactiae*** |  |  |  |  |
| Asia/Pacific Rim |  | n=713 | |  |
|  | LVX | 1 | 92.4 | p<0.001 |
| Latin America |  | n=1,132 | |  |
|  | MIN | ≥16 | 27.9 | p<0.01 |
| ***Streptococcus pneumoniae*** | |  |  |  |
| Asia/Pacific Rim |  | n=985 (859)^a^ | |  |
|  | AMC | 4 | 86.9 | p<0.0001 |
|  | AZM | ≥128 | 45.6 | p<0.001 |
|  | CRO | 2 | 86.5 | p<0.0001 |
|  | CLR | ≥128 | 45.8 | p<0.001 |
|  | CLI | ≥128 | 59.8 | p<0.0001 |
|  | ERY | ≥128 | 45.4 | p<0.001 |
|  | MIN | ≥16 | 36.2 | p<0.0001 |
| Europe |  | n=5,834 | |  |
|  | AMC | 1 | 96.3 | p<0.001 |
|  | CRO | 1 | 96.5 | p<0.0001 |
|  | MIN | 8 | 59.4 | p<0.0001 |
| Latin America |  | n=1,171 | |  |
|  | AMC | 2 | 95.0 | p<0.01 |
|  | CRO | 1 | 94.6 | p<0.001 |
|  | MIN | 8 | 57.0 | p<0.0001 |
|  | PEN | 2 | 54.6 | p<0.01 |
| Middle East |  | n=461 (429)^a^ | |  |
|  | AZM | 64 | 69.7 | p<0.01 |
|  | CLR | 64 | 69.5 | p<0.01 |
|  | ERY | 64 | 69.5 | p<0.01 |
|  | MIN | ≥16 | 44.5 | p<0.0001 |
| North America |  | n=5,887 (5,188)^a^ | |  |
|  | AMC | 2 | 90.4 | p<0.0001 |
|  | AZM | 64 | 64.8 | p<0.0001 |
|  | CRO | 1 | 95.5 | p<0.0001 |
|  | CLR | 64 | 65.2 | p<0.0001 |
|  | CLI | ≥128 | 83.1 | p<0.01 |
|  | ERY | 64 | 64.3 | p<0.0001 |
|  | MIN | 8 | 76.7 | p<0.0001 |
|  | PEN | 2 | 59.0 | p<0.0001 (+) |
| Global |  | n=14,562 (12,973)^a^ | |  |
|  | AMC | 2 | 92.8 | p<0.0001 |
|  | CRO | 1 | 95.0 | p<0.0001 |
|  | CLI | ≥128 | 80.8 | p<0.001 |
|  | MIN | 8 | 64.4 | p<0.0001 |
|  | PEN | 2 | 61.9 | p<0.0001 (+) |
| ***Streptococcus pneumoniae***, PRSP | |  |  |  |
| Africa |  | n=63 | |  |
|  | CRO | 2 | 85.7 | p<0.01 |
| Asia/Pacific Rim |  | n=296 | |  |
|  | AMC | 8 | 58.4 | p<0.001 |
|  | CRO | 4 | 56.4 | p<0.0001 |
|  | MIN | ≥16 | 9.5 | p<0.0001 |
| Europe |  | n=601 | |  |
|  | AMC | 8 | 66.2 | p<0.0001 |
|  | CRO | 2 | 71.5 | p<0.0001 |
|  | MIN | ≥16 | 25.5 | p<0.0001 |
| Latin America |  | n=167 | |  |
|  | CRO | 2 | 67.7 | p<0.01 |
|  | MIN | ≥16 | 31.7 | p<0.0001 |
| Middle East |  | n=114 (107)^a^ | |  |
|  | AZM | 64 | 38.3 | p<0.01 |
|  | CLR | 64 | 38.3 | p<0.01 |
|  | ERY | 64 | 38.3 | p<0.01 |
|  | MIN | ≥16 | 24.6 | p<0.01 |
| North America |  | n=911 (829)^a^ | |  |
|  | AMC | 8 | 39.6 | p<0.0001 |
|  | AZM | ≥128 | 16.0 | p<0.01 |
|  | CRO | 2 | 72.9 | p<0.0001 |
|  | CLR | ≥128 | 16.4 | p<0.01 |
|  | CLI | ≥128 | 40.9 | p<0.001 |
|  | ERY | ≥128 | 15.8 | p<0.01 |
|  | MIN | ≥16 | 33.8 | p<0.0001 |
| Global |  | n=2,152 (1,971)^a^ | |  |
|  | AMC | 8 | 53.2 | p<0.0001 |
|  | CRO | 2 | 69.9 | p<0.0001 |
|  | CLI | ≥128 | 43.2 | p<0.01 |
|  | MIN | ≥16 | 27.6 | p<0.0001 |

Imipenem significance results are not presented where n<50.

MRSA, methicillin-resistant *S. aureus*; PRSP, penicillin-resistant *S. pneumoniae*; VR, vancomycin-resistant.

^a^ The n values in parentheses represent the numbers of isolates tested against macrolides + clindamycin

^b^ Changes in significance indicated by (+) represent increased susceptibility; all others represent decreased susceptibility. A cut-off of p<0.01 was used for statistical significance testing.
